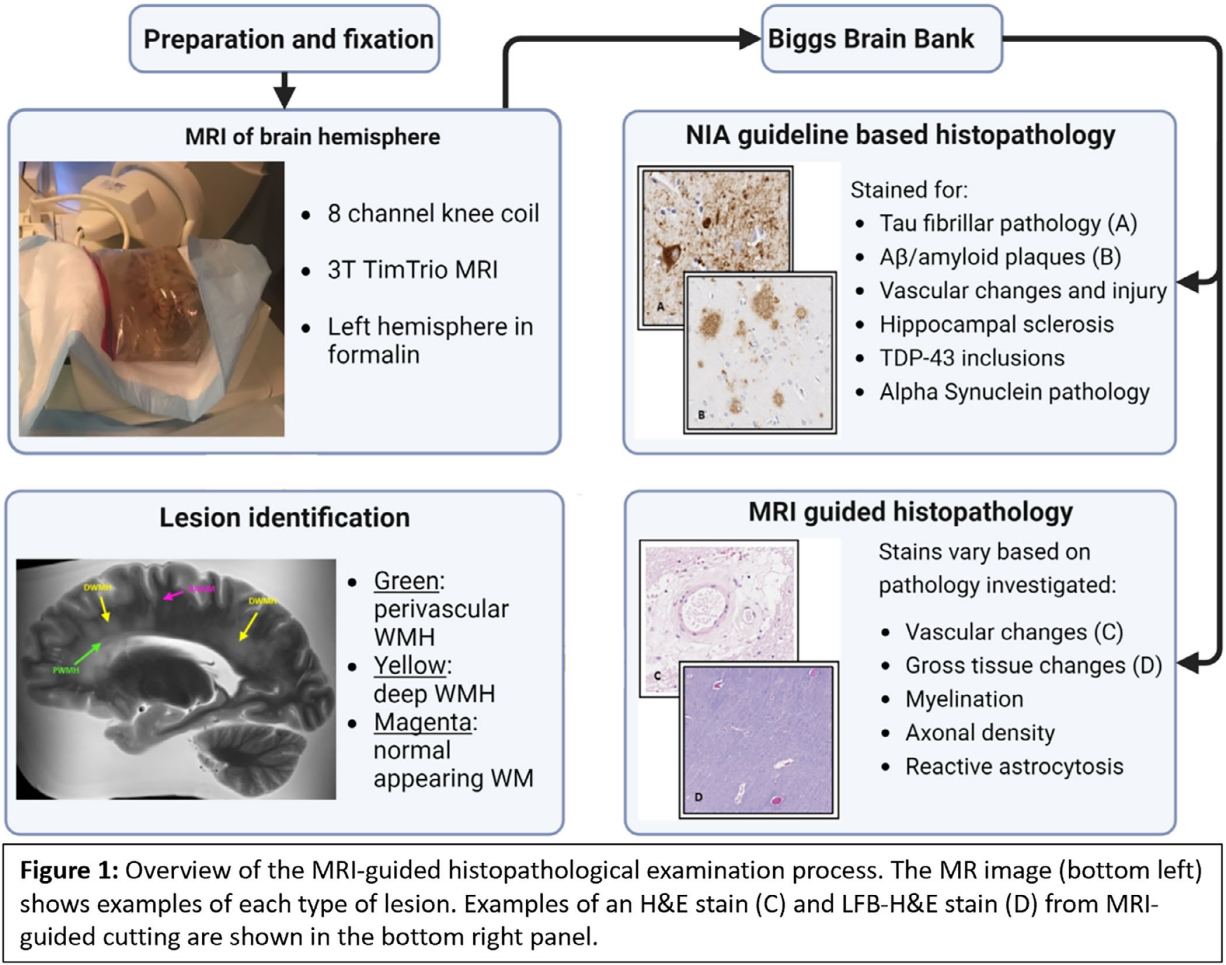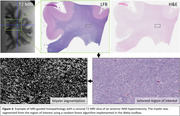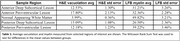# Spatial heterogeneity in white matter hyperintensities revealed by postmortem MRI‐guided histopathological staining

**DOI:** 10.1002/alz.093061

**Published:** 2025-01-09

**Authors:** Mariam Mojtabai, Karl Li, Tanweer Rashid, Jinqi Li, Nicolas Honnorat, Anoop Benet Nirmala, David M Martinez, David H Wang, Sokratis Charisis, Crystal Franklin, Mallory Keating, Marco I Boisselier, Haritha V. Katragadda, Gladys E. Maestre, Margaret E Flanagan, Tim Richardson, Jamie M. Walker, Peter T Fox, Sudha Seshadri, Kevin F. Bieniek, Mohamad Habes

**Affiliations:** ^1^ Glenn Biggs Institute for Alzheimer’s & Neurodegenerative Diseases, University of Texas Health Sciences Center at San Antonio, San Antonio, TX USA; ^2^ Research Imaging Institute, University of Texas Health Science Center at San Antonio, San Antonio, TX USA; ^3^ Department of Pathology and Laboratory Medicine, University of Texas Health Science Center at San Antonio, San Antonio, TX, USA., San Antonio, TX USA; ^4^ University of Texas Rio Grande Valley School of Medicine, Brownsville, TX USA; ^5^ Institute of Neuroscience at the University of Texas Rio Grande Valley, Harlingen, TX USA; ^6^ Icahn School of Medicine at Mount Sinai, New York, NY USA; ^7^ Department of Pathology and Laboratory Medicine, University of Texas Health Science Center at San Antonio, San Antonio, TX USA

## Abstract

**Background:**

White matter (WM) hyperintensities are bright areas on T2 MRI that reflect increased interstitial fluid caused by demyelination and axonal loss; these tissue alterations have been associated with cognitive impairment. Previous *in‐vivo* studies have suggested that the underlying pathogenesis for WM changes differs between the anterior and posterior brain, with cerebrovascular disease contributing more to anterior WM lesions and neurodegenerative processes contributing more to posterior WM lesions.

**Method:**

Periventricular (PV) and deep subcortical (DS) WM hyperintensities both in the anterior and posterior portions of the brain were identified using postmortem T2 MRI of cerebral hemispheres from the Biggs Institute Brain Bank (Figure 1) in 7 Alzheimer’s Disease patients (four male, three female, average age 75). Normal appearing (NA) WM at the base of the postcentral gyrus was selected as an internal control. Slide sections were obtained from these five regions across seven brains for 35 slides total using MRI landmarks to guide brain sectioning for histopathological analysis. The slides were stained using Luxol fast blue (LFB) and hematoxylin and eosin (H&E) and scanned digitally. Myelin was measured with the LFB, and vacuolation was measured with the H&E. Machine learning methods were used to segment and analyze the slides as the percent area stained in a selected region of interest (Figure 2).

**Results:**

Compared to NA WM, areas of WM hyperintensities exhibited a lower average LFB‐stained area (p=0.001) and H&E‐stained area (p<0.001), indicating increased demyelination and vacuolation within the lesions. Anterior PV WM lesions demonstrated a higher extent of demyelination (p=0.055) and vacuolation (p=0.074) than posterior PV WM lesions (Table 1). Posterior DS WM lesions showed significantly more demyelination than posterior PV WM lesions (p=0.022).

**Conclusion:**

The variations in demyelination within the WM lesions suggest differing underlying pathogenesis. This may be mediated by the differences in contribution from vascular and neurodegenerative causes as confirmed in previous studies. These preliminary results encourage the use of additional histopathological methods to better understand how different heterogeneous etiologies contribute to the development of white matter damage and how this can be translated into prevention strategies.